# Using an anterolateral thigh flap in autologous breast reconstruction as a salvage procedure in a patient with adult-onset Still’s disease: A case report

**DOI:** 10.3205/iprs000171

**Published:** 2023-03-02

**Authors:** Tarek Al-Malat, Berivan Taskin, Sebastian Schäller, Daniela Mettal-Minski, Lijo Mannil

**Affiliations:** 1 Department of Plastic and Aesthetic Surgery, St. Vinzenz Hospital, Cologne, Germany; 2 Department of Rheumatology, Helios Klinik Attendorn, Germany

**Keywords:** anterolateral thigh flap, breast reconstruction, deep inferior epigastric artery perforator flap, AOSD, interdisciplinary

## Abstract

The deep inferior epigastric artery perforator (DIEP) flap is an excellent option for microsurgical breast reconstruction. In selected cases, e.g. in case of previous abdominoplasty, other autologous options like transverse upper gracilis (TUG) or superior gluteal artery perforator (sGAP) flaps can be considered. The anterolateral thigh (ALT) flap is reported to be used as a salvage procedure in selected cases of breast reconstruction, where other flaps were not available or failed. We present a case of a 41-year-old woman who was undergoing bilateral breast reconstruction after bilateral mastectomies following implant-based mastopexie and multiple infections. She also suffered from an adult onset Still’s disease (AOSD) and was thus immunosuppressed. Microsurgical breast reconstruction was performed in a two-stage procedure. The left breast was reconstructed using a TUG flap. On the right side the TUG reconstruction failed due to vascular anomaly, so an ALT flap was successfully used instead. The whole procedure was accompanied by a multidisciplinary approach including a rheumatological complex treatment and enabled a successful bilateral breast reconstruction in this challenging case.

## Abstract (de)

Die DIEP- (deep inferior epigastric artery perforator) Lappenplastik gilt als Goldstandard in der mikrochirurgischen Brustrekonstruktion. In ausgewählten Fällen, wie zum Beispiel nach vorangegangener Bauchdeckenstraffung, können andere Eigengewebstransfers wie die TMG- (transverse myokutane Gracilislappenplastik) oder sGAP- (superior gluteal artery perforator) Lappenplastik erwogen werden. Die ALT- (anterolateral thigh) Lappenplastik ist als Rückzugsoption in wenigen Fällen der Brustrekonstruktion beschrieben, wenn andere Lappenplastiken nicht verfügbar waren oder nach Scheitern einer initialen mikrochirurgischen Brustrekonstruktion. Wir präsentieren den Fall einer 41-jährigen Patientin mit bilateraler mikrochirurgischer Brustrekonstruktion nach bilateraler Mastektomie in Folge einer implantatbasierten Augmentationsmastopexie und multipler Infektionen. Sie litt zudem an einem adulten Morbus Still und war daher immunsupprimiert. Im Rahmen einer zweizeitigen bilateralen Brustrekonstruktion wurde zunächst die linke Brust durch eine TMG-Lappenplastik von rechts rekonstruiert. Die Rekonstruktion der rechten Brust durch eine geplante TMG-Lappenplastik von links war aufgrund einer vaskulären Variante nicht möglich, sodass eine Rekonstruktion durch eine ALT-Lappenplastik erfolgte. Die gesamte Behandlung wurde interdisziplinär von einer rheumatologischen Komplexbehandlung begleitet und nur so war eine erfolgreiche bilaterale Brustrekonstruktion in diesem herausfordernden Fall möglich.

## Case report

We present a case of a 41-year-old woman desiring autologous bilateral breast reconstruction following bilateral mastectomies after implant removal following multiple infections. She experienced massive weight loss (–50 kg) due to a change of lifestyle. She also suffered from adult-onset Still’s disease (AOSD).

### Adult-onset Still’s disease (AOSD)

AOSD is an uncommon inflammatory condition of unknown origin typically characterized by four main (cardinal) symptoms: spiking fever, arthralgia or arthritis, skin rash, and hyperleucocytosis. It is also associated with the Koebner phenomenon, which describes the appearance of new skin lesions on previously unaffected skin secondary to trauma [1].

AOSD is rare. Its incidence is reported in a range between 0.16 and 0.4/100,000 people and estimated prevalence rate between 1 and 34 cases/1 million people. It is considered to be equally distributed between genders and usually affects young people with peaks at ages 15–25 and 36–46 years. It is generally accepted that unknown factors, acting as second hit, may trigger a pathologic process in genetically susceptible patients, finally leading to the activation of an aberrant inflammatory response [2].

Growing evidence is also showing a correlation between silicone breast implants and many forms of autoimmune diseases, whereas AOSD displays a very rare subtype [3].

Described disease courses are variable. There are monocyclic/self-limited courses (defined as a single episode (2 months to 1 year) followed by sustained remission), polycyclic/intermittent courses (with recurrent systemic flares between remissions) and – most frequently – chronic courses (at least one episode of persistent symptoms lasting longer than 1 year) [4].

The first line therapy for AOSD is based on corticosteroids. In the presence of corticosteroid resistance or dependence, conventional disease-modifying anti-rheumatic drugs (DMARDs) should be used, especially methotrexate [5].

For refractory disease, biological therapy with agents blocking interleukin-1 (anakinra) and those blocking interleukin-6 (tocilizumab) seem the most promising [6]. 

Several other severe complications have been reported in AOSD, including disseminated intravascular coagulopathy (DIC) or thrombotic thrombocytopenic purpura (TTP). Microangiopathic discorders like retinal microangiopathy are described in literature [7]. Although there is no evidence in literature for microsurgical procedures being affected by AOSD, we assume that these findings may have an influence on microsurgical free flap transfer in these selected patients.

### Patient’s history and interdisciplinary treatment

**Figure 1:**
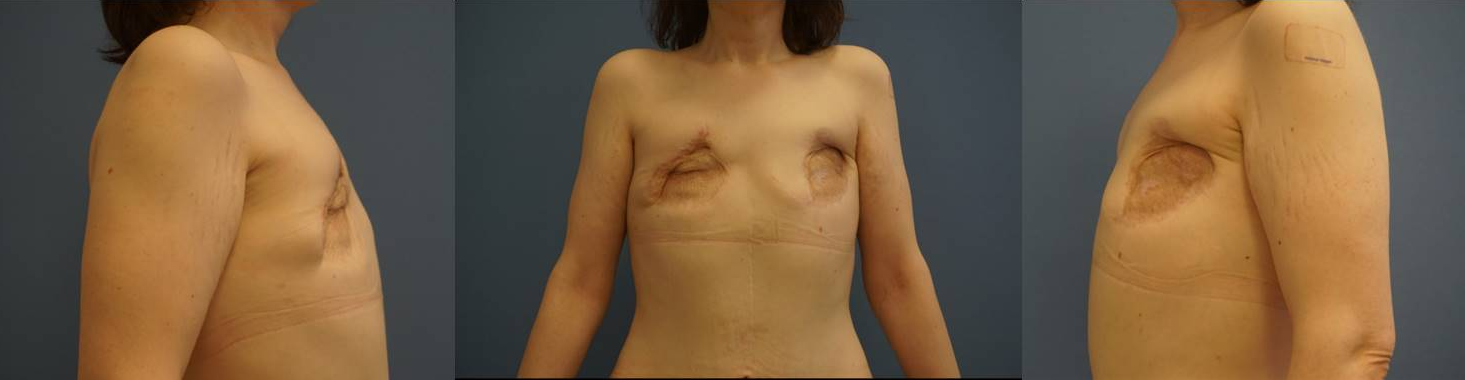
Preoperative view of the patient in an anterior view (middle) and laterally (left, right) demonstrating the adherent mesh graft scars on both sides

The patient presented with meshgraft scars on both sides of the ventral thorax (Figure 1 (Fig. 1)). Functionally, there was resulting thorax rigidity, and because of her painful scars, she was limited in taking a deep breath. In the past, she had visited many reconstructive surgeons, but no one had offered a reconstructive procedure because of her complicated course up to that point. 

Her surgical history included multiple operations after massive weight loss: Liposuction of both thighs years before and, furthermore, a Fleur-de-Lis abdominoplasty in 2015, followed by an implant-based augmentation mastopexy in 2016. Implant removal on the right side was necessary due to a hematoma and subsequent infection two months later. After seven months with multiple debridements, episodes of recurrent fever, and delayed wound healing, a radical mastectomy on the right side was performed. Histologically, there was no evidence for a presumed pyoderma gangrenosum, but there was for an unspecific perivascular issue and interstitial dermatitis. The resulting defect on the thoracic wall was covered with a Meshgraft procedure from the right thigh. Wound healing was delayed again, and a second Meshgraft procedure was performed. 

After a few months, implant removal on the left side was necessary due to infection with a subsequent mastectomy. An episode of multiple debridements followed on the left breast, and in the end, a Mesh-graft procedure was again required to cover the defect. During that time, the patient was also psychiatrically examined to exclude other reasons for recurrent infections and disrupted wound healing (e.g., Munchausen syndrome). There was no evidence of a psychiatric disorder. Like on the right side, a second Mesh-graft coverage of the defect on the left side was necessary because of severe wound healing and infection issues. Complete wound healing on both sides was not yet seen.

Because of this course with many complications, an intensive rheumatological examination was performed. During a two-week complex rheumatological treatment with intravenous cortisone therapy in combination with intravenous immunoglobulin (IVIG), the wounds finally healed by secondary intention. In the further course, the patient was treated with an interleukin-1 beta receptor antagonist (canakinumab).

When we examined the patient the first time, there was no possibility to reconstruct her breast from abdominal tissue after she had undergone an abdominoplasty in the past.

**Figure 2:**
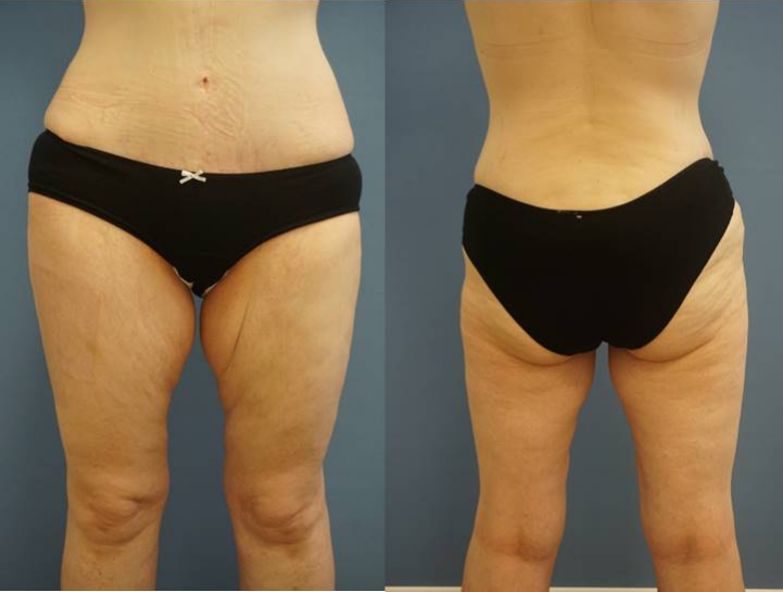
Preoperative view of the patient anterior and posterior: after previous abdominoplasty a DIEP flap was not possible, but there was substantial tissue on both thighs for breast reconstruction.

Usage of the deep inferior epigastric perforator flap (DIEP flap) as the considered gold-standard of microsurgical breast reconstruction was not possible [8]. She presented with substantial tissue on both medial thighs, so we recommended an autologous reconstruction with a transverse upper gracilis (TUG) flap (Figure 2 (Fig. 2)). Because of her surgical history, we decided to reconstruct one breast after the other. We consulted the rheumatological specialist and discussed the patient’s reconstructive desires. The specialist recommended 20 g of IVIG one week before the operation and 10 g of IVIG once a week after the operation until wound healing was completed. Further medication included Prednisolon 70 mg per day and Canakinumab 2x 150 mg every 3 weeks.

### Microsurgical breast reconstruction 

Subsequently, we reconstructed her left breast with a free TUG flap from the right thigh. The adherent skin graft was removed from the chest wall, and the recipient internal mammary vessels were prepared for microsurgical anastomosis. An end-to-end anastomosis was performed between the flap vessels and the left internal mammary artery and vein. The recipient site healed well; the donor site wound on the right medial thigh developed delayed wound healing that was first treated with dressings and intensive wound care. After 14 days, a surgical revision of the donor site was necessary due to wound dehiscence and little seroma formation. Bacterial contamination could not be detected microbiologically. The donor site wound then healed delayed but without further surgical intervention. The patient was ambulated after 25 days in good physical condition. 

**Figure 3:**
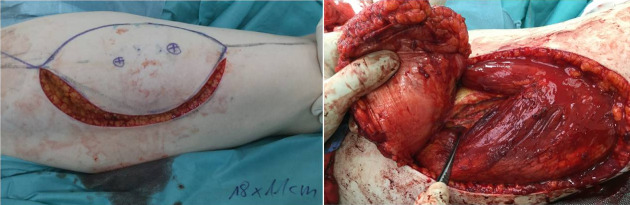
Left: lateral incision during ALT flap raise, two perforators marked preoperatively. Right: demonstrating the undersurface of the ALT flap in the subfascial plane with forceps pointing on the pedicle.

After six months, we prepared to reconstruct her right breast with a free TUG flap from the left thigh using the same preoperative medical treatment strategy. Surprisingly, there was no proximal dominant pedicle supplying the gracilis muscle. Instead, the proximal pedicle was leading into the adductor longus muscle. This course of the gracilis pedicle is not uncommon [9]. Converting it into a profunda artery perforator flap was not possible because there were no suitable perforators to ensure skin island perfusion. The decision was made to suture the elevated anterior half of the skin island back in place. After discussing possible reconstructive procedures with the patient, we decided to offer an anterolateral (ALT) flap for reconstruction. Doppler determination revealed two perforating vessels on the right thigh. One week later, an 18 cm x 11 cm skin island was designed in a vertical ellipse on the right thigh, and incision was carried down through the fascia in a subfascial plane. A large septocutaneous perforator was proximally explored arising from the descending branch of the lateral femoral circumflex artery (Figure 3 (Fig. 3)). The second distal perforator was small and thus severed after temporary clipping with a Biemer clip to make sure perfusion was guaranteed via the proximal perforator. The flap was then harvested on this single perforator, and an end-to-end anastomosis was performed between the flap vessels and the internal mammary artery and vein. The flap had excellent perfusion and was inset. Doppler examination confirmed adequate perfusion. The donor site was closed primarily after placing two suction drains. Postoperatively, the ALT donor site on the right healed primarily, and the donor site on the left medial thigh showed wound dehiscence proximally and was treated with debridements and intermittently with vacuum-assisted closure therapy. A secondary wound closure was possible, but the patient again developed delayed wound healing, which was treated with dressings and wound care. The patient was ambulated after 32 days. The intravenous immunoglobulin therapy was then stopped.

**Figure 4:**
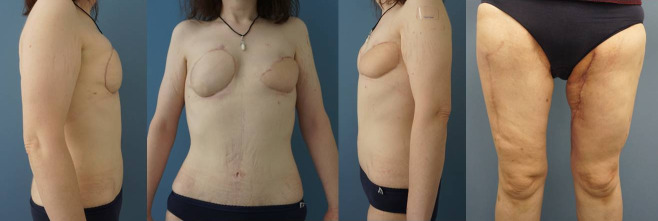
Six months postoperatively: breast reconstruction with TUG flap (left breast) and ALT flap (right breast) in an anterior and lateral view. Donor site on both thighs in an anterior view (right).

Six months later, she underwent thigh lifts in combination with liposuction. In this setting, the former skin island of the left TUG flap was removed, and lipofilling to achieve upper pole fullness in both neo-breasts was performed (Figure 4 (Fig. 4)). The patient is scheduled for the next lipofilling in three to six months. After the desired volume is achieved and refinements are completed, reconstruction of the nipple-areolar complex will be planned.

### The anterolateral thigh flap

First described by Song in 1984 [10], the ALT flap has become a versatile flap in microsurgical reconstruction. It is commonly used as a free flap in head and neck reconstruction [11], [12] and soft tissue defects in upper [13] and lower [14] extremity reconstruction. It can also be used as a pedicled flap for defect coverage (e.g., in the abdominal wall [15] or perineal defects [16]).

#### Anatomy

The ALT flap, in most cases, is supplied by perforators arising from the lateral circumflex femoral artery, which originates from the profunda femoral artery or femoral artery. It divides into ascending, transverse, and descending branches. The descending one runs obliquely between the vastus lateralis and intermedius muscles and sends perforators to supply the skin island overlying the flap. Sensation of the flap is possible and provided by the lateral femoral cutaneous nerve (L2-3). When designing the flap, most perforators are found inferolaterally in a circle of 3 cm diameter in the midline between the anterior superior iliac spine and the lateral border of the patella. Doppler examination is essential in flap design, which is mostly drawn as a longitudinal ellipse with the perforator located in the proximal third [17].

#### Usage in breast reconstruction

The ALT flap’s use in breast reconstruction was first described by Wei et al. in 2002 in five cases of postmastectomy reconstruction [18]. Another case report including three cases was reported by Kaplan et al. in 2003 [19]. It has also been reported as a salvage procedure following failed deep inferior epigastric perforator flap reconstruction in two cases [20]. Its usage in bilateral breast reconstruction has been reported [21] as well as in unilateral large breast reconstruction using bilateral ALT flaps [22]. All of these cases included patients with a history of breast cancer, and the ALT flap was used in cases where other donor sites were not available for different reasons. 

To our knowledge, there has not been a case reported in a non-breast-cancer patient with AOSD to date. 

### Conclusion

Rheumatological examination and treatment with intravenous immunoglobulins were essential in this case as invasive treatments with surgical debridements were not addressing the underlying AOSD. The multidisciplinary approach of plastic surgeons and rheumatologists finally enabled a successful bilateral breast reconstruction in this significant problem case.

## Notes

### Competing interests

The authors declare that they have no competing interests.

### Patient consent

Written informed consent for the publication of this case report and of the images was obtained from the patient.
